# Impact of respiratory infection in the results of cardiac surgery in a tertiary hospital in Brazil

**DOI:** 10.5935/1678-9741.20150038

**Published:** 2015

**Authors:** Isaac Newton Guimarães Andrade, Diego Torres Aladin de Araújo, Fernando Ribeiro de Moraes

**Affiliations:** 1 Universidade Federal de Pernambuco (UFPE), Recife, PE, Brazil.; 2 Estudante de Medicina da Universidade Federal de Pernambuco (UFPE), Recife, PE, Brazil and fellow of the Programa Institucional de Bolsas de Iniciação Científica (Pibic), Recife, PE, Brazil.; 3 Universidade Federal de São Paulo (Unifesp), São Paulo, SP, Brazil.

**Keywords:** Bronchopneumonia, Thoracic Surgery, Postoperative Care

## Abstract

**Objective:**

To assess the impact of respiratory tract infection in the postoperative period of cardiac surgery in relation to mortality and to identify patients at higher risk of developing this complication.

**Methods:**

Cross-sectional observational study conducted at the Recovery of Cardiothoracic Surgery, using information from a database consisting of a total of 900 patients operated on in this hospital during the period from 01/07/2008 to 1/07/2009. We included patients whose medical records contained all the information required and undergoing elective surgery, totaling 109 patients with two excluded. Patients were divided into two groups, WITH and WITHOUT respiratory tract infection, as the development or respiratory tract infection in hospital, with patients in the group without respiratory tract infection, the result of randomization, using for the pairing of the groups the type of surgery performed. The outcome variables assessed were mortality, length of hospital stay and length of stay in intensive care unit. The means of quantitative variables were compared using the Wilcoxon and student t-test.

**Results:**

The groups were similar (average age *P*=0.17; sex *P*=0.94; surgery performed *P*=0.85-1.00) Mortality in the WITH respiratory tract infection group was significantly higher (P<0.0001). The times of hospitalization and intensive care unit were significantly higher in respiratory tract infection (P<0.0001). The presence of respiratory tract infection was associated with the development of other complications such as renal failure dialysis and stroke *P*<0.00001 and *P*=0.002 respectively.

**Conclusion:**

The development of respiratory tract infection postoperative cardiac surgery is related to higher mortality, longer periods of hospitalization and intensive care unit stay.

## Abstract (pt)

**Objetivo:**

Avaliar o impacto da infecção do trato respiratório no pós-operatório da cirurgia cardíaca no Hospital Português de Pernambuco em relação à mortalidade hospitalar e identificar os pacientes com maior risco de desenvolver essa complicação.

**Métodos:**

Estudo do tipo transversal observacional realizado na Unidade de Recuperação de Cirurgia Cardiotorácica, utilizando informações de um banco de dados composto por um total de 900 pacientes operados nesse hospital no período de 01/07/2008 a 31/07/2009. Foram incluídos pacientes cujos prontuários continham todas as informações necessárias, totalizando 109 pacientes, havendo exclusão de dois. Os pacientes foram divididos em dois grupos, com e sem infecção do trato respiratório, conforme o desenvolvimento ou não infecção do trato respiratório no internamento, sendo os pacientes do grupo sem infecção do trato respiratório, fruto de randomização, utilizando-se para o pareamento dos grupos o tipo de cirurgia realizada. As variáveis de desfecho avaliadas foram mortalidade, tempo de internamento hospitalar e tempo de internamento em unidade de terapia intensiva. As médias das variáveis quantitativas foram comparadas por meio do teste de Wilcoxon e t de student.

**Resultados:**

Os grupos mostraram-se semelhantes. A mortalidade no grupo RTI foi significativamente maior (*P*<0,0001). Os tempos de internamento hospitalar e em unidade de terapia intensiva foram significativamente maiores no grupo infecção do trato respiratório (*P*<0,0001). A presença de infecção do trato respiratório associou-se ao desenvolvimento de outras complicações como insuficiência renal dialítica e acidente vascular cerebral, *P*<0,00001 e *P*=0,002, respectivamente.

**Conclusão:**

O desenvolvimento de infecção do trato respiratório no pós-operatório de cirurgia cardíaca relaciona-se a maior mortalidade, maiores tempos de internamento e permanência em unidade de terapia intensiva.

**Table:** 

**Abbreviations, acronyms & symbols**	
EuroSCORE	European System for Cardiac Operative Risk Evaluation
ICU	Intensive care unit
RFD	Renal failure dialysis
RHP	Real Hospital Português de Beneficiência
RTI	Respiratory tract infection
TSRU	Thoracic surgery recuperation unit

## INTRODUCTION

In Brazil, more than 100,000 heart surgeries are performed each year^[1]^. In Recife, at Real Hospital Português de Beneficência in Pernambuco (RHP) only, 1,400 surgeries are performed every year, demonstrating the significance of the procedure in our country. Many patients develop complications that affect the results, increasing morbidity and mortality at the individual level, and burdening the health care system. In heart surgery, three major events, when present, increase the chances of death: the development of respiratory infection, perioperative stroke and renal failure dialysis (RFD)^[2,3]^, Besides associated with higher mortality, such occurrences are important causes of readmission to the intensive care unit, increasing hospital costs^[4]^.

An important determinant of bad results in cardiac surgery is the infection^[5]^, especially the respiratory infection, the most frequent in this type of procedure - it exceeds 50% of high mortality infections^[6]^. It is also known that the early identification of patients at higher risk of developing this complication and the adoption of prophylactic measures can reduce the mortality rate significantly^[7-9]^.

The lack of local studies to determine the prevalence and the impact of renal tract infection (RTI) justifies this study. The understanding of this problem in our region can help implement intervention strategies that change the current situation.

### Objective

The aim of this study is to assess the impact of respiratory tract infection in cardiac surgery postoperative period in RHP, especially regarding the hospital mortality, and to identify patients at higher risk of developing this complication.

## METHODS

We used a cross sectional observational study that was made by the Thoracic Surgery Recuperation Unit (TSRU) of RHP.

Data were collected from existing database containing information of 900 patients operated and admitted to the TSRU of RHP, from July 1^st^ 2008 to July 31^st^ 2009.

The sample was initially composed by 109 patients, being two later excluded because of lack of data. The 107 remaining patients were divided into 02 groups: one defined as RTI Group, composed by 29 patients who developed respiratory tract infection, and Control Group, made up of 78 patients without RTI.

The variable used to pair the two groups was the type of performed surgery. Thus the type of surgery proportion performed on the RTI group was determined. Then there was a randomization among patients who did not develop RTI and were part of the database, thus forming the control group, with 78 patients.

For the RTI diagnosis, we used clinical respiratory infection parameters associated with tracheal aspirate secretion culture, with colony counts equal to or greater than one million units.

The sample size calculation was based on the RTI development prevalence in cardiac surgery postoperative, existing in the literature.

Demographic variables were assessed, such as gender and age, in addition to the type of surgery performed and the outcome variables, such as mortality, length of hospital stay and ICU length of stay. Categorical variables were expressed by their absolute and relative frequencies and the quantitative variables were expressed by their average and standard deviations.

To compare the averages of different groups, we used the t-test when the variables followed a normal distribution. For variables that did not follow this distribution, we used non-parametric tests (Wilcoxon). For association studies, we used the chi-square test or Fisher's exact when indicated.

When the alternative hypothesis was sought, *P*<0.05 were considered statistically significant.

BioStat 5.0 was used.

The project was submitted to the Ethics Committee of RHP and to the Ethics Research Committee of UFPE. We requested authorization for the use of the records in this study.

## RESULTS

The sample was composed by 107 patients, divided into 2 groups: with and without RTI.

After pairing the groups, it was observed that they had no difference in age average (*P*=0.17), gender distribution (*P*=0.94) and type of performed surgery (*P*=0.85-1.00), demonstrating the similarity of the population of the two groups.

A higher average value of the EuroSCORE was observed in the group WITH RTI, compared to the control group, (WITHOUT RTI), with a tendency to statistical significance (*P*=0.07).

We found a significantly higher mortality in the group with RTI (48% *vs*.3.8%), as shown in Table 1.

**Table 1:** Respiratory tract infection association with renal failure dialysis and stroke.

RFD*		Stroke**	
Groups	%	Groups	%
Group with RTI	31	Group with RTI	17
Group without RTI	0	Group without RTI	0

* P<0.00001 Fisher’s Exact;

** P=0.002 Fisher’s Exact;

RTI=respiratory tract infection; RFD=renal failure dialysis

**Fig. 1:**
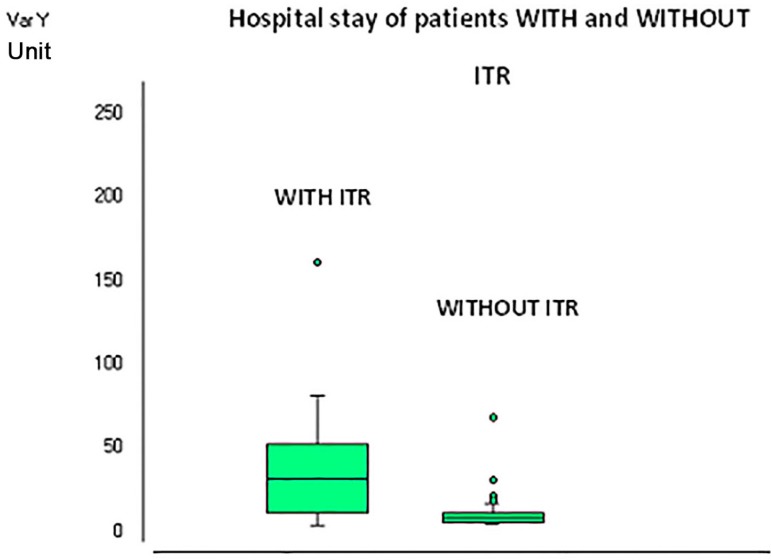
Hospitalar stay in the two groups.

**Fig. 2:**
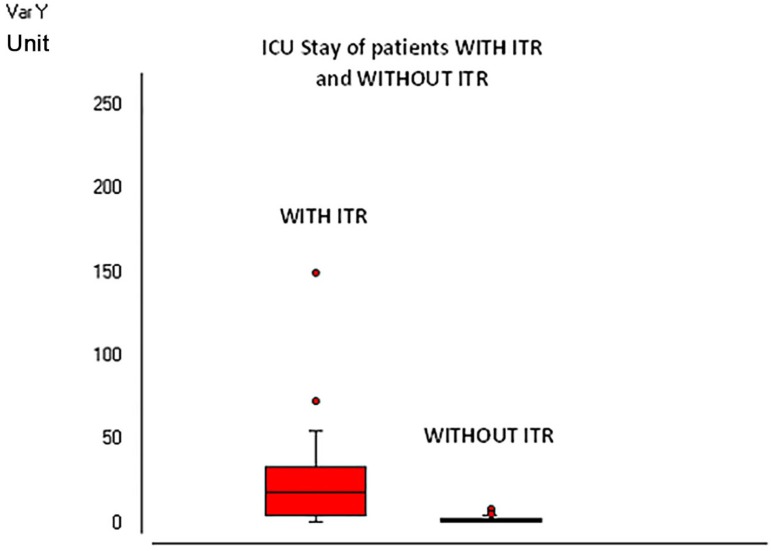
Intensive care unit stay in the two groups.

The length of hospital stays and ICU length stay was significantly higher in the group WITH RTI compared to the control group (*P*<0.0001 and *P*=0.002 respectively), as Figures 1 and 2 show.

In the group with RTI an important association of RTI with the development of other complications, such as renal failure dialysis (RFD) and stroke (*P*<0.0001 and *P*=0.002 respectively). This association was not observed in the control group (Table 1).

Studying the RTI group specifically and analyzing the risk group by EuroSCORE separately (high, medium and low), we found no statistically significant association between risk group and prevalence of RTI. However, dividing them in low and medium-high, we found a higher prevalence of RTI on medium-high of patients, compared to low risk (*P*=0.01).

### Chance to develop high and medium risk RTI compared to low risk.

The chance high and medium risk patients had develop RTI was four times higher than low risk patients (Table 2).

**Table 2:** Prevalence of respiratory tract infection in RTI group and chance to develop RTI, according to EuroSCORE risk group.

RTI group*		EuroSCORE group**	
Groups	N	Risk Group	OR
Group low risk	6	Low risk	1.0
Group medium-high risk	23	High-medium risk	3.83 ( IC 95% =1.36 - 10.79)

* P=0.01;

* P=0.016;

RTI=respiratory tract infection; OR=odds ratio.

## DISCUSSION

Analysis showed the similarity between control groups and disease, the result of proper pairing. No differences were observed between the analyzed groups in relation to gender distribution, age average and performed surgery. Pairing by type of surgery performed eliminated an important bias, since most complex surgeries tend to have higher prevalence of complications^[10-12]^.

A second bias that could sully the results would be the inappropriate use of perioperative antibiotics, or patients who have been operated in the presence of respiratory infection. However all patients enrolled in the study underwent elective surgery and therefore evaluated before discarding infection prior surgery. All patients were subjected to the same antibiotic scheme.

The RTI group had higher EuroSCORE^[13]^ average compared to no RTI, with a tendency to statistical significance. That is, higher prevalence of RTIs in high-risk patients, which confirms the results of studies that associate higher risk (such as age, diabetes and kidney disease) to the higher prevalence of complications^[14-16]^.

Mortality in patients with RTI was significantly higher than in the group without RTI, as demonstrated in the literature^[17,18]^.

The length of hospital stay and the time of ICU stay were significantly higher in the group with RTI compared to the control group. This fact implies probably a higher cost, as intensive care units (ICUs) make up about 20% of total hospital costs^[19]^. Data from this study, in line with published data, show that the existence of complications after heart surgery is directly related to a longer hospital stays and higher mortality rate^[20]^.

Cardiac surgery has as primarily non-cardiac complications the development of infection (most respiratory), IRA and stroke^[21]^. This was observed in this study, and it is interesting to note the fact that the RFD and stroke are most commonly associated with respiratory infection. That is, the RTI is strongly associated with such complications. Failure to observe the presence of RFD and stroke in the group without RTI reinforces this claim.

## CONCLUSION

The development of RTI in cardiac surgery postoperative is related to higher mortality, as well as to longer hospital and ICU stay. This complication has also been associated with the development of other co-morbidities such as renal failure dialysis and stroke.

**Table:** 

**Authors’ roles & responsibilities**	
INGA	Analysis and/or interpretation of data; Statistical analysis study design;
DTAA	Performed operations and/or experiments
FRMN	Final approval of the manuscript
